# Prevalence, determinants and health care-seeking behavior of childhood acute respiratory tract infections in Bangladesh

**DOI:** 10.1371/journal.pone.0210433

**Published:** 2019-01-10

**Authors:** Marufa Sultana, Abdur Razzaque Sarker, Nurnabi Sheikh, Raisul Akram, Nausad Ali, Rashidul Alam Mahumud, Nur Haque Alam

**Affiliations:** 1 Nutrition and Clinical Services Division, International Centre for Diarrheal Disease Research, Bangladesh (icddr,b), Dhaka, Bangladesh; 2 School of Health and Social Development, Deakin University, Burwood, Melbourne, Australia; 3 Health Systems and Population Studies Division, International Centre for Diarrheal Disease Research, Bangladesh (icddr,b), Dhaka, Bangladesh; 4 University of Strathclyde, Glasgow, United Kingdom; 5 University of Southern Queensland, Queensland, Australia; University of Dhaka, BANGLADESH

## Abstract

**Background:**

Acute respiratory infections (ARIs) are one of the leading causes of child mortality worldwide and contribute significant health burden for developing nations such as Bangladesh. Seeking care and prompt management is crucial to reduce disease severity and to prevent associated morbidity and mortality.

**Objective:**

This study investigated the prevalence and care-seeking behaviors among under-five children in Bangladesh and identified factors associated with ARI prevalence and subsequent care-seeking behaviors.

**Method:**

The present study analyzed cross-sectional data from the 2014 Bangladesh Demographic Health Survey. Bivariate analysis was performed to estimate the prevalence of ARIs and associated care-seeking. Logistic regression analysis was used to determine the influencing socio-economic and demographic predictors. A p-value of <0.05 was considered as the level of significance.

**Result:**

Among 6,566 under-five children, 5.42% had experienced ARI symptoms, care being sought for 90% of affected children. Prevalence was significantly higher among children < 2 years old, and among males. Children from poorer and the poorest quintiles of households were 2.40 (95% CI = 1.12, 5.15) and 2.36 (95% CI = 1.06, 5.24) times more likely to suffer from ARIs compared to the wealthiest group. Seeking care was significantly higher among female children (AOR = 2.19, 95% CI = 0.94, 5.12). The likelihood of seeking care was less for children belonging to the poorest quintile compared to the richest (AOR = 0.03, 95% CI = 0.01, 0.55). Seeking care from untrained providers was 3.74 more likely among rural residents compared to urban (RRR = 3.74, 95% CI = 1.10, 12.77).

**Conclusion:**

ARIs continue to contribute high disease burden among under-five children in Bangladesh lacking of appropriate care-seeking behavior. Various factors, such as age and sex of the children, wealth index, the education of the mother, and household lifestyle factors were significantly associated with ARI prevalence and care-seeking behaviors. In addition to public-private actions to increase service accessibility for poorer households, equitable and efficient service distribution and interventions targeting households with low socio-economic status and lower education level, are recommended.

## Background

Acute respiratory infections (ARIs) remain an important public health concern that is widely recognised as the major cause of mortality and morbidity among under-five children [1]. Globally, one-fifth of mortality among under-five children is attributable to ARIs, predominantly pneumonia, which is particularly responsible for 18% of total under-five deaths [2,3]. ARIs continue to persist as the single largest morbidity contributor among children, and is responsible for approximately 70% of under-five childhood morbidities in developing countries [2]. Approximately 3.5% of the global disease burden is caused by ARIs, and are responsible for 30% to 50% of total pediatric outpatient visits and up to 30% of pediatric admissions in developing countries [2–4]. In 2004, the World Health Organization (WHO) and the United Nations (UN) suggested that ARIs should be addressed as ‘presumed pneumonia’ [3]. Annually, approximately 150 million childhood pneumonia episodes are reported worldwide with an estimated 1.3 million deaths each year while 90% of these deaths occur in developing countries [4]. Despite a global reduction of under-five mortality of nearly half from 1990 to 2012 [3], under-five mortality caused by pneumonia remains higher compared to child deaths caused by other preventable infectious diseases and post-neonatal situations [5,6,7].

Bangladesh has achieved Millennium Development Goals (MDGs) 4 targets by reducing the under-five mortality rate by 74% from 1990 to 2015 [8]. Despite this notable improvement, Bangladesh remained in the top ten countries with the greatest number of under-five deaths in 2015, contributing 2% of the total under five deaths globally [9] and remaining in the top 15 countries child deaths prevalence due to pneumonia and diarrhea [10]. The prevalence of ARIs among children under-five years of age in Bangladesh was 39.8 per 1000 children in 2013; however, Hib conjugate vaccine was introduced into the national immunization program in Bangladesh since 2009 [11,12]. ARIs were also accountable for about 39% of total pediatric hospital admissions and, 40 to 60% of total pediatric outpatient department visits in Bangladesh [13]. With a mission of health system strengthening, the health system of Bangladesh has made primary and secondary health care services available in both rural and urban areas [14,15]. Despite this improvement, seeking care from health facilities or medically trained provider remains low (42%) [8]; therefore, Bangladesh continues to bear a high ARI-related mortality burden (21%) regardless of the relative reduction of other infectious diseases [8,16].

It was observed that numerous socio-economic and other factors including age of the child, household income, household condition, parental education, maternal age and other factors are associated with ARI [1,3,17–19]. Gender-based discrimination and characteristics of the caregivers have been shown to generate differences in the pattern of utilization of health care services in Africa and Asia [20,21]. Although ARI still remains as a public health concern in Bangladesh, limited studies focused on prevalence and associated factors of ARI using nationwide recent survey data.

A study was conducted approximately 10 years ago based on 2004 BDHS round in which ARI risk factors were identified [18]. Furthermore, Bangladesh achieved remarkable improvements in all of the health-related Millennium Development Goals (MDGs), and also set new goals for improving child health as per the more recent Sustainable Development Goals (SDGs). Current evidence using nationally representative data on household care-seeking behavior and determinants considering ARI remain scarce. In order to improve health the care system through increasing accessibility and affordability, it is essential to expand our understanding of the current prevalence, associated determinants and related health care seeking regarding ARIs. As such, this study aims to determine the prevalence of ARIs in under-five children in Bangladesh and to identify associated determinants and care-seeking behaviors.

## Materials and methods

### Data and study population

The present study utilized nationally representative cross-sectional data from the more recent Bangladesh Demographic and Health Survey (BDHS) 2014. The survey was implemented from June to mid-November 2014, under the National Institute of Population Research and Training (NIPORT) of the Ministry of Health and Family Welfare [8]. The survey design adopted a multi-staged stratified sampling technique to capture relevant information on maternal and child health (MCH) including childhood morbidity and healthcare-seeking practices that enabled the estimation of MCH indicators representative at national and divisional levels [8,22]. The detailed description of methods, design, instruments, participants and sampling frame had previously been published by NIPORT [8]. A structured questionnaire was administered by trained and experienced interviewers, and 17,863 ever-married reproductive (15–49 years) women were interviewed with a 98% response rate to capture socio-economic, demographic, and health care utilisation data at the household level [8]. There are seven separate datasets in the BDHS, and, we created a single merged dataset using household and child identifiers and analyses were drawn from the merged dataset. To elicit ARIs specific information, mothers were asked to provide information on the history of cough accompanied by short, rapid chest-related breathing and/or difficulty in breathing among children aged 0–59 months in the two weeks prior to the survey. According to the WHO, the presence of these symptoms is defined as “suspected pneumonia”, and the survey considered the symptoms of ARI as a proxy measure of pneumonia [22,23].

### Ethical approval

BDHS 2014 is a publicly available dataset and can be downloaded from the DHS Program website (https://dhsprogram.com/data/available-datasets.cfm). We analyzed the dataset after receiving approval from the MEASURE DHS program office. The survey followed standardized data collection procedures and received ethics approval from the National Research Ethics Committee (NREC) of the Bangladesh Ministry of Health and Family Welfare. According to the DHS, written informed consent was obtained from all participants before they enrolled in the survey.

### Outcome variables

The outcome variables included ‘symptoms of acute respiratory infections (ARIs)’, and ‘mother sought care for their child with ARI symptoms’. The presence of any ARI symptoms was coded as “1”, while the absence of symptoms was coded as “0”. Cases with the presence of ARI symptoms were specifically considered for further analysis. The second outcome variable was coded as “1” if care was sought and “0” if no care was sought. Care seeking behavior was further categorized as; ‘no care’, ‘pharmacy’, ‘public care’, ‘private care’, and ‘traditional care’ according to other published literature using the same dataset [24]. Public care included care sought from any trained providers in a public facility such as any hospital (district, medical college, and specialized), Maternal and Child Welfare Centre (MCWC), Upazila Health Complex (UHC), Union Health & Family Welfare Centre (UH&FWC), satellite clinic/EPI outreach site, or Community Health Care Providers (CHCP). Similarly, NGO static clinics, NGO satellite clinics, NGO fieldworkers, private hospital/clinics, and qualified doctors were included in private care. Care provided by unqualified/untrained personnel without any formal medical training such as traditional healers, self-management at home, and using herbals for remedy were incorporated into the ‘traditional care’.

### Major explanatory variables

Major explanatory variables included the nutritional index of the child, maternal and paternal education level, parental occupation, drinking water source, sanitary facilities, household size, crowding, floor and roof materials, cooking condition, fuel used, geographical distribution, and wealth index of the households. The Nutritional status of children was measured using standard child physical growth indices according to the WHO by generating z-scores for ‘height-for-age (stunting)’, ‘weight-for-height (wasting)’ and ‘weight-for-age (underweight)’ [8,24]. According to WHO criterion, children were considered stunted, wasted, or underweight if the z-score for each nutritional status lay ‘two standard deviations below the median of the WHO reference population [8,24]. Maternal occupation was classified as housewife/no occupation/unemployed, poultry raising/farming/cultivation (land owner, poultry/cattle raising and home based manufacturing), business/semi-skilled & unskilled labor (small business/trader, brick breaking, domestic servant and factory worker), professional/business/technical (doctor, lawyer, large business, dentist, accountant and other service holder). Similarly, paternal occupation was classified as unemployed, agricultural (land owner, farmer, and poultry), semi-skilled and unskilled (small business/trader, brick breaker, and factory worker), small business/traders and professional/business/technical accordingly.

According to DHS, household socioeconomic status was measured by calculating the wealth index using principal component analysis (PCA) to assign the ad hoc weights of the indicators. This is a combined measure of the cumulative living standard and calculated by using selected household’s assets through generating a factor score as a weight. The procedure includes calculating factor coefficient scores and standardizing indicator variables. Finally, indicator values were multiplied by the factor loadings to generate the index value per household [25]. For this subset analysis, the socioeconomic status of households having under-five children was measured by calculating the wealth index as per the DHS guideline and categorized into the ‘poorest’, ‘poorer’, ‘middle’, ‘richer’ and ‘richest’ quintiles [8]. Other categorizations of the explanatory variables were determined by published studies on childhood illness in developing countries including Bangladesh based on BDHS survey [24,26,27]. Drinking water source was classified as “improved” in case of water piped into the dwelling, piped to the yard/plot, public tap/standpipe, tube-well or borehole, protected well, rainwater, bottled water. Drinking water source was classified as “not-improved” if it consisted of an unprotected well, unprotected spring, tanker truck/cart with drum, or surface water. Similarly, toilet facilities were defined as “improved” in case of flush/pour flush to piped sewer system, flush/pour flush to septic tank, flush/pour flush to pit latrine, ventilated improved pit (VIP) latrine, and pit latrine with slab. Toilet facilities were considered “not-improved” in the case of flush/pour flush not to sewer/septic tank/pit latrine, pit latrine without slab/open pit, hanging toilet/hanging latrine, or no facility/bush/field [26]. Household floor types were categorized as earth/sand and others (wood planks, palm, bamboo, ceramic tiles, cement, and carpet). Household density was defined as “crowded” if three or more people slept in one room and “not-crowded” otherwise. Fuel type for cooking was categorized as “clean fuel” in case of using electricity, LPG gas, natural gas, and biogas, whereas the “polluted fuel” category included the use of kerosene, coal, lignite, charcoal, wood, straw/grass/shrubs, agricultural crop, and animal dung.

### Statistical analysis

All of the 17,863 ever-married women with under- five children were included in the descriptive analysis. Further analysis was conducted for children with the ARI symptoms. Sampling weight provided by the BDHS database was used to weight the data. Descriptive bivariate analysis techniques (i.e. frequency distribution, cross tabulation, mean, standard deviation) were calculated to understand the prevalence of ARIs and healthcare-seeking behavior with 95% confidence intervals and differentiated with socio-demographic, economic, maternal, child and behavioral factors. To investigate the influential factors for childhood ARI symptoms and to address the factors influencing health care utilization, binary logistic regression analysis was performed and reported as an odds ratio (OR) with 95% CI for both adjusted and unadjusted models. Crude/unadjusted odds ratios were obtained by considering the effect of only one predictor variable, while adjusted odds ratios were obtained by including all influential variables in the model to take into account for the effect of all additional confounding variables included in the regression model. Finally, a multinomial multivariate logistic regression model was executed and results were presented in terms of adjusted RRR (relative risk ratio) with a 95% confidence interval. All statistical analyses were performed using the statistical package STATA (version 13), and results were interpreted as statistically significant at a p-value of <0.05.

## Results

### Socio-demographic characteristics

A total of 6,566 women with children aged <5 years were included in the study. The mean child age was 30 months, and 52% of children were male (Table 1). Approximately one-third of children were stunted and underweight, while 14% were wasted. Overall, 55% of mothers and 43% of fathers had completed secondary or higher education. Nearly all fathers but only 29% of mothers were currently employed. Most children lived in rural areas (74%), and average family size was five members per household. Electronic media access was available among 44% of households. Approximately 98% of households used drinking water from improved sources, while 67% had access to improved toilet facilities, and most household floors were earth/sand (69%). Half of the households (51%) were crowded, and 85% used polluted fuel for cooking, though only 15% cooked inside their living rooms.

**Table 1:** Distribution of socio-demographic characteristics of under-five children (N = 6,566).

Variable(s)	n (%)	95% CI
**Child Age (in month)**		
**Mean age (mean ± s.d)**	30.04 ± 16.92	(29.63, 30.64)
< 12 m	1,207 (18.38)	(17.07, 19.81)
12–23 m	1407 (21.43)	(20.45, 22.44)
24–35 m	1317 (20.05)	(19.10, 21.04)
36–47 m	1302 (19.83)	(18.89, 20.82)
48–59 m	1333 (20.31)	(19.35, 21.30)
**Sex of Child**		
Male	3,416 (52.02)	(50.81, 53.23)
Female	3,150 (47.98)	(46.77, 49.19)
**Nutritional Status**		
**Height for Age**		
Normal	4,176 (63.60)	(62.43, 64.76)
Stunted	2,390 (36.40)	(35.24, 37.57)
**Weight for Height**		
Normal	5,621 (85.60)	(84.73, 86.43)
Wasted	945 (14.40)	(13.57, 15.27)
**Weight for Age**		
Normal	4,412 (67.20)	(66.05, 68.32)
Underweight	2,154 (32.80)	(31.68, 33.95)
**Presence of ARI symptom**		
Yes	356 (5.42)	(4.90, 6.00)
No	6,210 (94.58)	(94.00, 95.10)
**Mean age in year (mean ± sd)**	25.78 ± 5.90	(25.64, 25.93)
Less than 20	886 (13.50)	(12.69, 14.35)
20–34	5,143 (78.32)	(77.31, 79.30)
35+	537 (8.18)	(7.54, 8.87)
**Mother’s Education level**		
No education	1,126 (17.15)	(16.26, 18.08)
Primary	1,843 (28.07)	(26.99, 29.17)
Secondary & Higher	3,597 (54.78)	(53.58, 55.98)
**Mother’s Occupation**		
Housewife/no occupation	4,653 (70.87)	(69.76, 71.96)
Poultry raising/farming/cultivation	1,117 (17.02)	(16.13, 17.94)
Business/semi-skilled & unskilled labor	389 (5.93)	(5.38, 6.53)
Professional/technical	406 (6.18)	(5.63, 6.79)
**Father’s Education level**		
No education	1,748 (26.63)	(25.57, 27.71)
Primary	1,992 (30.34)	(29.24, 31.47)
Secondary	1,955 (29.78)	(28.69, 30.90)
Higher	870 (13.25)	(12.45, 14.09)
**Father’s Occupation**		
Unemployed	61 (0.93)	(0.72, 1.19)
Agricultural/farming	1,753 (26.69)	(25.63, 27.77)
Semi-skilled and unskilled labor	2,952 (44.96)	(43.76, 46.17)
Small business/traders	1,280 (19.5)	(18.56, 20.47)
Professional/large business/technical	520 (7.92)	(7.29, 8.60)
**Household size**		
Up to five members	3,697 (56.31)	(55.11, 57.51)
Above five members	2,869 (43.69)	(42.49, 44.89)
**Mother’s access to electronic media**		
Yes	2,901 (44.17)	(42.98, 45.38)
No	3,666 (55.83)	(54.62, 57.02)
**Source of drinking water**		
Improved	6,418 (97.75)	(97.36, 98.08)
Non-improved	148 (2.25)	(1.92, 2.64)
**Type of toilet**		
Improved	4,387 (66.81)	(65.67, 67.94)
Non-improved	2,179 (33.19)	(32.06, 34.33)
**Type of floor**		
Earth/sand	4,544 (69.20)	(68.07, 70.31)
Other floors	2,022 (30.80)	(29.69, 31.93)
**Household crowding characteristics**		
Not crowded (<3 persons sleep in a room)	3,221 (49.06)	(47.85, 50.27)
Crowded (≥ 3 persons sleep in a room)	3,345 (50.94)	(49.73, 52.15)
**Household fuel types**		
Clean fuel	1,017 (15.49)	(12.85, 14.54)
Polluted fuel	5,549 (84.51)	(85.46, 87.15)
**Place of cooking**		
Inside	1,017 (15.48)	(14.63, 16.38)
In a separate building	4,509 (68.67)	(67.54, 69.78)
Outside	1,040 (15.84)	(14.98, 16.75)
**Residence**		
Urban	1,689 (25.73)	(24.68, 26.80)
Rural	4,877 (74.27)	(73.20, 75.32)
**Wealth Index**		
Poorest	1,509 (22.98)	(21.98, 24.02)
Poorer	1,225 (18.65)	(17.73, 19.61)
Middle	1,277 (19.45)	(18.51, 20.43)
Richer	1,305 (19.88)	(18.93, 20.86)
Richest	1,250 (19.03)	(18.10, 20.00)

### ARI Prevalence and health care-seeking behavior

**Fig 1:**
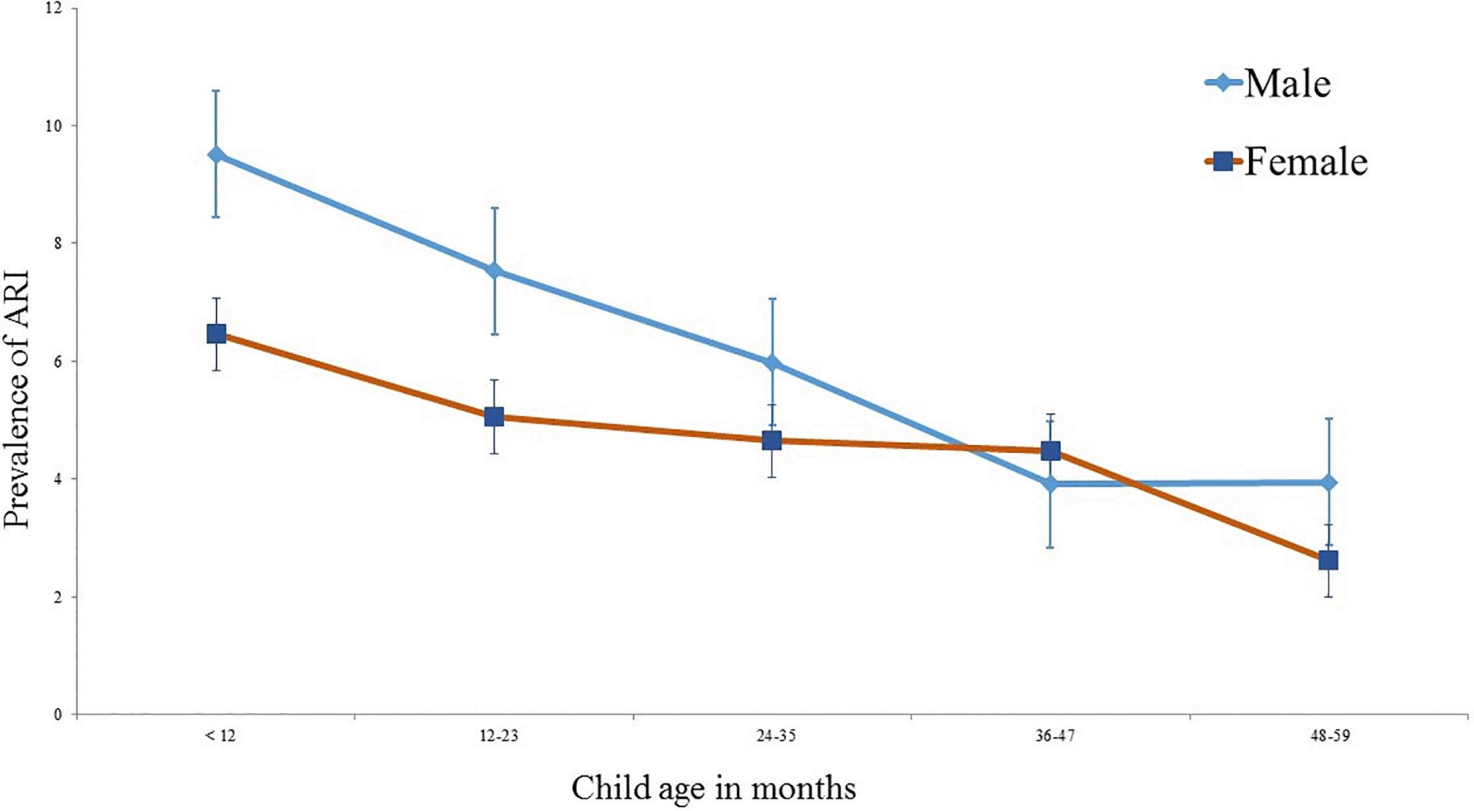
Prevalence of ARI among under-five children by different age groups.

According to the responses of mothers, 356 (5.42%) children had the defined symptoms of ARI in the two weeks prior to the survey. There was a gradual decline of prevalence with increasing child age, with the highest occurrence (8.11%) being observed among children aged <12 months, and the lowest at 48–59 months (3.29%) (Table 2). Prevalence was higher among male children (6.19%) compared to female (4.60%) children; however, although this gender difference varied by age, with a similar prevalence observed at older ages (Fig 1).

**Table 2:** Prevalence and factors associated with childhood ARI in Bangladesh (N = 6,566).

Variables	Prevalence of ARI, n (%)	Model-I	Model-II
		Unadjusted OR (95%CI)	Adjusted OR (95%CI)
**Child Age (in month)**			
< 12 m	98 (8.11)	2.59*** (1.80, 3.73)	2.55*** (1.75, 3.72)
12–23 m	90 (6.37)	2.00*** (1.38, 2.89)	1.93*** (1.32, 2.81)
24–35 m	70 (5.33)	1.65** (1.13, 2.43)	1.71** (1.16, 2.52)
36–47 m	55 (4.19)	1.28 (0.86, 1.93)	1.29 (0.86, 1.94)
48–59 m (ref)	44 (3.29)	1.00	1.00
**Sex of Child**			
Male	211 (6.19)	1.37** (1.10, 1.70)	1.25** (1.02, 1.59)
Female (ref)	145 (4.60)	1.00	1.00
**Nutritional Status**			
**Height for Age**			
Normal	201 (4.82)	1.00	-
Stunted	155 (6.48)	1.37** (1.08, 1.70)	-
**Mother’s age (in year)**			
**Mean age (mean ± sd)**			
Less than 20	72 (8.13)	1.69*** (1.29, 2.22)	1.32** (1.02, 1.76)
20–34 (ref)	256 (4.98)	1.00	1.00
35+	28 (5.24)	1.05 (0.71, 1.57)	1.24 (0.82, 1.88)
**Mother’s Education level**			
No education	49 (4.36)	0.93 (0.67, 1.28)	0.67 (0.45, 0.99)
Primary	138 (7.51)	1.65*** (1.31, 2.08)	1.26** (1.01, 1.65)
Secondary & Higher (ref)	169 (4.69)	1.00	1.00
**Mother’s Occupation**			
Housewife/no occupation (ref)	240 (5.15)	1.00	1.00
Poultry raising/farming/cultivation	57 (5.09)	0.99 (0.73, 1.33)	0.93 (0.68, 1.26)
Business/semi-skilled & unskilled labor	36 (9.31)	1.89*** (1.31, 2.72)	2.05*** (1.40, 3.02)
Professional/technical	23 (5.76)	1.13 (0.73, 1.74)	1.39 (0.88, 2.18)
**Father’s Education level**			
No education	98 (5.60)	2.58*** (1.58, 4.22)	1.92** (1.09, 3.38)
Primary	132 (6.62)	3.08*** (1.90, 4.99)	2.23*** (1.31, 3.78)
Secondary	107 (5.46)	2.51*** (1.54, 4.10)	2.11*** (1.27, 3.50)
Higher	20 (2.25)	1.00	1.00
**Mother’s access to electronic media**			
Yes	127 (4.38)	1.00	1.00
No	229 (6.25)	1.45*** (1.16, 1.82)	0.99 (0.73, 1.35)
**Type of toilet**			
Improved (ref)	219 (4.99)	1.00	1.00
Non-improved	137 (6.29)	1.28** (1.03, 1.59)	1.04 (0.81, 1.34)
**Type of floor**			
Earth/sand	268 (5.90)	1.37** (1.07, 1.76)	0.70 (0.44, 1.12)
Other floors (ref)	88 (4.36)	1.00	1.00
**Household crowding characteristics**			
Not crowded (<3 persons sleep in a room) (ref)	153 (4.75)	1.00	1.00
Crowded (≥ 3 persons sleep in a room)	203 (6.07)	1.30** (1.05, 1.61)	1.11 (0.88, 1.41)
**Household fuel types**			
Clean fuel (ref)	40 (3.97)	1.00	1.00
Polluted fuel	316 (5.69)	1.46** (1.04, 2.04)	1.04 (0.65, 1.65)
**Place of cooking**			
In the house	66 (6.54)	1.42*** (1.07, 1.88)	1.74*** (1.29, 2.35)
In a separate building (ref)	212 (4.71)	1.00	1.00
Outdoors	77 (7.45)	1.63*** (1.24, 2.13)	1.45** (1.10, 1.92)
**Residence**			
Urban (ref)	75 (4.44)	1.00	1.00
Rural	281 (5.77)	1.32** (1.01, 1.71)	1.13 (0.82, 1.55)
**Wealth Index**			
Poorest	99 (6.53)	2.17*** (1.48, 3.16)	2.36** (1.06, 5.24)
Poorer	81 (6.60)	2.19*** (1.48, 3.24)	2.40** (1.12, 5.15)
Middle	69 (5.43)	1.78*** (1.19, 2.66)	2.07** (1.03, 4.13)
Richer	68 (5.23)	1.71*** (1.15, 2.56)	1.75** (1.07, 2.86)
Richest (ref)	39 (3.12)	1.00	1.00

**p<0.05

***p<0.001

Model 1 shows crude odds ratio of the individual predictor variable on the prevalence of ARI

Model 2 shows adjusted model by including all the predictor variables to take into account the effect of all confounding variables on the prevalence of ARI

Notably, the prevalence of ARI symptoms appeared to be higher among malnourished children (Table 2). Moreover, the prevalence of ARI symptoms was associated with maternal age, maternal and paternal education, and parental occupation (Table 2). ARI symptom prevalence was also higher in rural households (compared to urban) and in households of lower wealth and lower resources (compared to those of higher wealth and resources).

### Factors associated with ARI symptoms

Adjusted logistic regression indicated that child age, gender, maternal age, parental education, occupation, socioeconomic status, and household cooking area were significantly associated with the presence of ARI symptoms. Significant associations were also observed with the residence of the household, media access, toilet type, floor type, crowding characteristics, and cooking fuel type in the unadjusted model. The odds of having ARI symptoms were highest among youngest children (aged <12 months) and it was 2.59 higher compared to reference age. Moreover, male children were 1.25 times more likely to suffer from ARI symptoms than female children. Importantly, the economic status of households was significantly associated with ARIs prevalence; in particular, children from the poorer and poorest households were more likely to have ARI symptoms. The other factors with a significant impact on ARIs prevalence included young mothers, less parental education, the mother being occupied with semi-skilled or unskilled labor, and the cooking practice of the households. However, no significant differences were observed among nutritional status, the residence, crowding, and access to electronic media, drinking water source, toilets type, and cooking fuels in relation to the adjusted model.

**Fig 2:**
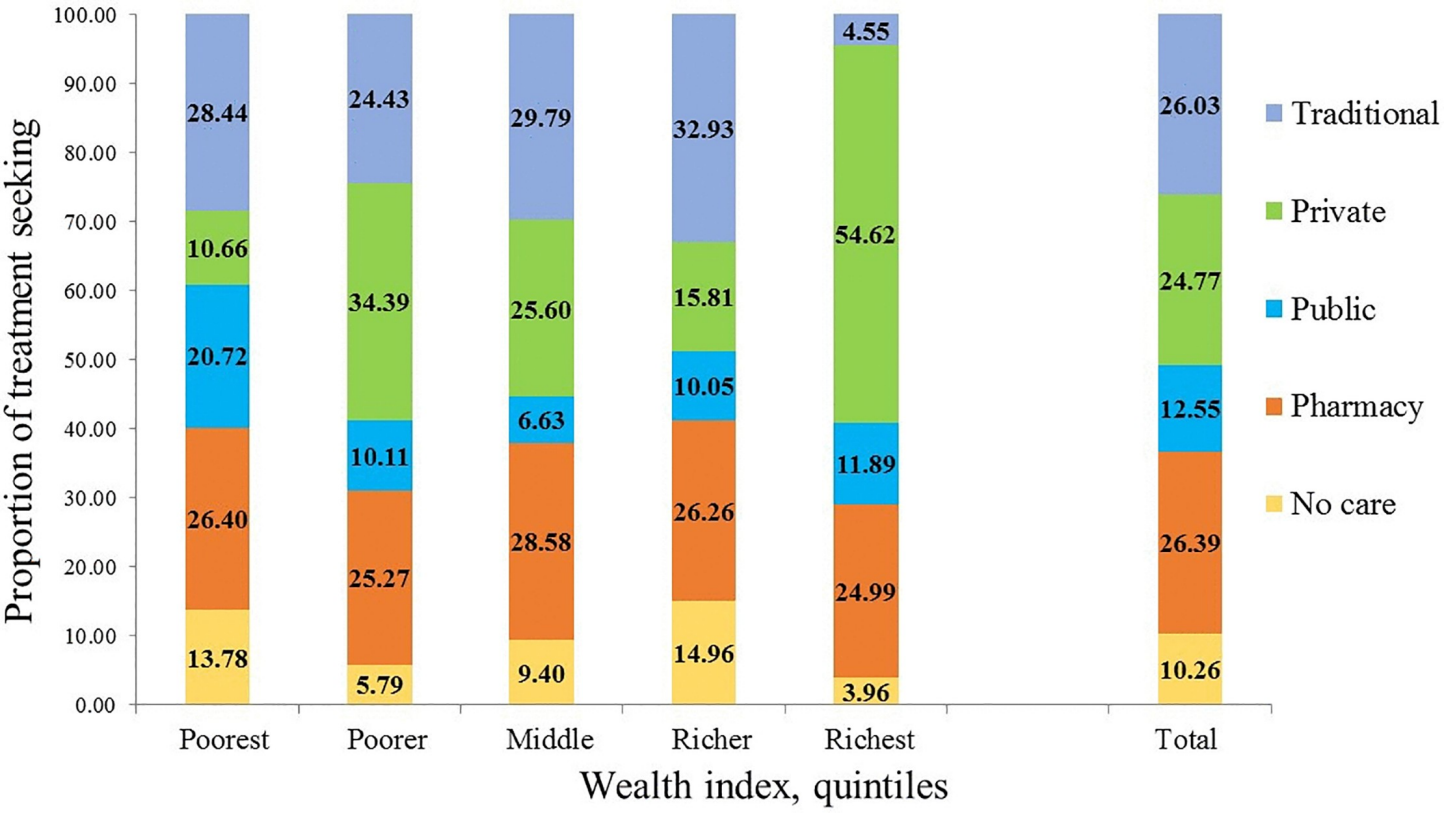
Proportion of treatment seeking behavior for childhood ARI among different economic groups and by different facilities.

Our results suggest that, most children (90%) received some type of care for ARI symptoms. Overall, 26.39%, 12.55%, 24.77%, and 26.03% utilized care from pharmacies, public facilities, private facilities, and traditional health care providers respectively. Households in the poorest quintile were most likely to use public facilities and least likely to use private facilities (Fig 2). Households in the richest quintile were most likely to use private facilities and least likely to use traditional services. However, the overall pattern of service use varied across the wealth quintiles. Pharmacies were the source of about 25% of care sought by children with ARI symptoms, regardless of wealth (Fig 2).

### Determinants of health seeking behavior

Table 3 presents the factors associated with health care-seeking patterns due to presence of ARI symptoms among children. Three factors such as gender, economic status, and household floor type were found to be significantly associated with the utilization of healthcare for ARI. A higher probability of seeking care among caregivers was observed among females (AOR = 2.19). Moreover, the economic status of households was identified as an important predictor, with care from medical providers being sought by households who belonged to the richest quintile. Additionally, the findings revealed that the probability of receiving treatment from trained providers increased gradually with improving economic status. In addition, children who lived in a household with earth/sand floor types were less likely to seek care than the reference category. Overall, households were 5.80 times more likely to seek care for their youngest children from a public provider. Seeking care from the private facilities was more commonly observed among female children, while seeking care from private facilities was higher among working mothers. However, children living in rural areas were 3.74 times more likely to receive care from traditional providers than urban children.

**Table 3:** Factors associated with health seeking behaviour for childhood ARI in Bangladesh (n = 356).

Variables	Received any health care, n (%)	Binary logistic regression	Multivariate Multinomial logistic model^1^		
		Care vs No care	Public facility	Private facility	Traditional
		Adjusted OR (95% CI)	RRR^2^- (95% CI)	RRR^2^ (95% CI)	RRR^2^ (95% CI)
**Child Age (in month)**	** **	** **	** **	** **	
< 12 m	90 (92.29)	1.53 (0.41, 5.78)	5.80 (0.78, 43.33)	1.79 (0.44, 7.26)	0.94 (0.22, 4.00)
12–23 m	79 (88.40)	0.98 (0.28, 3.40)	4.05 (0.59, 27.63)	1.12 (0.30, 4.16)	0.46 (0.11, 1.88)
24–35 m	62 (87.58)	0.97 (0.26, 3.59)	3.99 (0.55, 29.07)	1.11 (0.28, 4.42)	0.5 (0.11, 2.17)
36–47 m	50 (91.30)	1.26 (0.30, 5.22)	1.66 (0.17, 16.66)	1.56 (0.35, 6.9)	0.75 (0.16, 3.55)
48–59 m (ref)	39 (88.30)	1.00	1.00	1.00	1.00
**Sex of Child**	** **				
Male (ref)	184 (86.85)	1.00	1.00	1.00	1.00
Female	136 (93.95)	2.19** (1.05, 5.12)	2.14 (0.71, 6.42)	2.53** (1.06, 6.04)	1.90 (0.74, 4.87)
**Mother’s age (in year)**	** **				
Less than 20	67 (93.41)	1.61 (0.30, 8.81)	0.24 (0.02, 2.59)	1.86 (0.32, 10.84)	1.87 (0.28, 12.38)
20–34	228 (89.05)	1.02 (0.26, 3.96)	0.60 (0.10, 3.66)	1.40 (0.34, 5.81)	0.57 (0.12, 2.71)
35+(ref)	24 (86.57)	1.00	1.00	1.00	1.00
**Mother’s Education level**	** **				
No education	41 (83.26)	0.73 (0.24, 2.23)	0.37 (0.07, 1.95)	1.12 (0.35, 3.64)	0.38 (0.10, 1.50)
Primary	124 (89.97)	1.12 (0.46, 2.70)	1.05 (0.33, 3.38)	1.19 (0.47, 2.98)	0.86 (0.32, 2.31)
Secondary & Higher (ref)	154 (91.43)	1.00	1.00	1.00	1.00
**Mother’s Occupation**	** **				
Housewife/no occupation (ref)	213 (88.84)	1.00	1.00	1.00	1.00
Poultry raising/farming/cultivation	53 (93.00)	1.91 (0.58, 6.31)	1.62 (0.34, 7.76)	1.32 (0.38, 4.62)	3.41** (1.01, 12.24)
Business/semi-skilled & unskilled labour	32 (88.58)	1.70 (0.49, 5.86)	5.45** (1.29, 23.05)	1.48 (0.41, 5.36)	0.33 (0.05, 2.26)
Professional/technical	22 (92.83)	0.87 (0.15, 5.17)	1.34 (0.17, 10.68)	0.85 (0.14, 5.29)	0.65 (0.09, 4.88)
**Mother’s access to electronic media**	** **				
Yes (ref)	114 (89.59)	1.00	1.00	1.00	1.00
No	206 (89.82)	1.14 (0.38, 3.38)	1.10 (0.23, 5.32)	1.09 (0.36, 3.37)	1.42 (0.41, 4.95)
**Type of floor**	** **				
Earth/sand	243 (90.61)	5.29** (1.02, 32.27)	3.75** (2.14, 15.64)	2.59 (0.36, 18.76)	2.67** (1.59, 10.78)
Other floors (ref)	77 (87.08)	1.00	1.00	1.00	1.00
**Household crowding characteristics**	** **				
Not crowded (<3 persons sleep in a room)	141 (91.89)	1.02 (0.43, 2.41)	0.49 (0.15, 1.64)	1.28 (0.53, 3.09)	0.83 (0.32, 2.18)
Crowded (≥ 3 persons sleep in a room) (ref)	179 (88.12)	1.00	1.00	1.00	1.00
**Residence**	** **				
Urban (ref)	64 (85.6)	1.00	1.00	1.00	1.00
Rural	255 (90.84)	2.05 (0.77, 5.45)	0.84 (0.22, 3.17)	1.71 (0.62, 4.74)	3.74** (1.10, 12.77)
**Wealth Index**	** **				
Poorest	85 (86.22)	0.03** (0.01, 0.55)	0.01** (0.02, 0.61)	0.03** (0.01, 0.78)	0.07 (0.01, 2.61)
Poorer	76 (94.21)	0.06 (0.02, 1.18)	0.02** (0.04, 0.87)	0.10 (0.02, 2.33)	0.11 (0.02, 3.98)
Middle	63 (90.60)	0.05** (0.01, 0.75)	0.01** (0.01, 0.32)	0.08 (0.01, 1.37)	0.13 (0.01, 3.29)
Richer	58 (85.04)	0.13** (0.02, 0.85)	0.09** (0.05, 0.96)	0.09** (0.01, 0.64)	1.06 (0.09, 12.50)
Richest (ref)	37 (96.04)	1.00	1.00	1.00	1.00

**p<0.05

***p<0.001

^1^ Reference group: No care

^2^ RRR = Relative Risk Ratio

## Discussion

This study aimed to explore the ARI prevalence, health-care seeking behaviors as well as their socio-demographic factors and socio-economic differentials using nationwide representative data from Bangladesh. It was observed that various socioeconomic and demographic factors, such as economic status, parental education, the age of the mother, residence, cooking habits, family size, and kitchen location were significantly associated with the prevalence and care-seeking behaviors related to ARI among children. Household wealth index was considered an important and significant determining factor for both ARI prevalence and care-seeking, which is inversely related to prevalence and positively associated with seeking appropriate health care for ARI symptoms.

The prevalence of ARI was 5.4% in this study which is similar to the previous two rounds of BDHS survey reports [28,29] and also similar to the overall prevalence in India, as reported by representative national data (5.8%) [28]. Considering the previous BDHS round, the prevalence of ARI was highest in 2004 (21%), then declined sharply and remained steady (5%) from 2007 until the current DHS. According to the DHS, care-seeking behavior from health care facilities was only 20% in 2004, which increased gradually to 37%, 35% and 42% for 2007, 2011 and 2014 respectively [8,28,29,30]. Comparatively lower prevalence was observed compared to the findings from other countries (22–40%), while a study based on 40 developing countries found average ARI prevalence of 13% [3,31,32]. This comparatively lower prevalence compared to other developing countries could be explained by the introduction of the Hib conjugate vaccine into the national immunization program and improvements to the health care system and infrastructure at the community level [11,33]. Furthermore, seasonal variation is another potential predictor s related to higher ARI prevalence. Several studies conducted in Bangladesh and other developing countries reported variation in ARI prevalence usually peaking during the winter and monsoon seasons [34–37]. This could be due to climates patterns and season length varying across different countries, which might be associated with varying ARI prevalence. Consistent with a previous study based on BDHS survey data in 2004, our study results also disclosed higher ARI prevalence among children aged <12 months, while male children were found to be more vulnerable to ARI symptoms [20]. Congruent with these findings, a wide range of studies in developing countries reported older age being associated with lower ARI prevalence, with children under 2 years of age being reported as most vulnerable [3,18,27,28]. These trends may be due to immature immune systems among young age groups or additional factors such as receiving care from different caregivers or playing with other peers potentially increasing infection risk at early ages [24,38]. Furthermore, in line with this study, a higher risk among male children was also reported in other studies, with boys being 7% more likely to develop ARIs [3,39]. The reason behind the high vulnerability of male children may be genetic, or there could be higher reporting for boys by the mothers due to gender bias, which potentially causes mothers to notice symptoms due to a higher preference for boys [40].

Children from adolescent mothers and malnourished children were also observed to be at higher risk of ARIs. Similar findings were also found in relevant studies conducted earlier [41–43]. This could be due to the negative health and well-being consequences resulting from teenage pregnancy. Additionally, household poverty may be a common influencing factor behind maternal age and poor nutrition, wherein poverty leads to early marriage among women, which subsequently that leads to early pregnancy, chronic malnourishment, a weakened immune system and, a subsequently increased vulnerability to disease. Most earlier studies revealed that household poverty, adolescent motherhood, and child malnourishment are significant ARI risk factors among children in Bangladesh [43,44]. Moreover, geographic region and socio-economic status were also identified as effective factors to ARI prevalence, and this study determined that the children from rural areas and the poorest communities were more prone to have ARI symptoms. These findings were also congruent with the findings of prior studies conducted in Bangladesh and other countries [3,10,20,21,23]. Women with primary education reported an increased frequency of ARI among their children, which is consistent with other study findings [23,45], though inconsistent with the findings reported by Pinzon et al. [3]. Higher reporting could be explained by the knowledge level of women who that able to recognize any ARI symptoms. Other contextual household lifestyle factors, such as cooking pattern, media access, floor materials, drinking water supply, and crowding were also found to be associated with ARI prevalence in this study. These factors are also explained by other studies as important factors for developing ARI symptoms, especially in the case of developing countries [43,46,47].

In the present study, nearly 90% of households sought care for their children from health care providers (including the traditional healers) upon encountering symptoms of ARI. Care-seeking behavior was relatively high compared to the combined findings reported from other developing countries (60%), and sub-Saharan Africa (48%)[19,48]. This is consistent with reports from other developing countries that indicated significant improvements in care seeking in recent years; however, the decision-making process to seek care is influenced by additional factors[17,19,49–50]. Relatively high levels of care seeking are inspiring in countries with low socio-economic development such as Bangladesh. The high percentage of care seeking might be explained by the availability of pharmacies, the establishment of various health centers (public, private, and NGO), improved health system infrastructure in recent years and even disease severity [17,19,51–52].

The type of care sought was significantly associated with wealth quintile, with parents from the highest quintile seeking care from trained providers (private or public facilities). These findings are also confirmed by earlier studies[19,23,24]. It is obvious that the cost of care plays a significant role in decision making -with out-of-pocket expenditures being the major share for receiving any care in the context of developing countries, as people from upper wealth quintiles are generally able to afford healthcare costs [19,53,54]. However, pharmacies were determined as the most common type of care sought by nearly all income quintiles. This could be due to self-medication practices with the availability of pharmacies in most areas of Bangladesh, where people can purchase medicine without prescriptions [55]. Additionally, in contrast to wealthiest group, the poorest and middle economic groups sought health care from the traditional providers, which is similar to findings from other recent studies [19,56]. This may be caused by the availability and low cost of these services, which can be accessed by poor communities.

Additional factors such as the age and sex of children, household type, parental education level, and the age of the mother significantly influenced care seeking patterns. Similar to the present study, an earlier study in Ethiopia observed high care seeking patterns among younger mothers[52]. Inconsistent with other studies, seeking care was 2.19 times more common for girls in our study while no gender differential was reported in others [20,57,58]. However, discrimination in care seeking against girls has been previously reported [59]. Higher levels of parental education significantly contributed to seek appropriate care for children in our study. It is evident that parental education significantly contributed to the seeking appropriate care and to the prevention and control of morbidity [19]. The evidence also suggests care-seeking behavior for children is higher among younger and more educated mothers in many low- and middle-income countries [58]. Similarly, another influencing factor is family size, as families with less members possibly allow parents to invest more time and money in their sick children [52].

### Methodological strength and limitations

The present study used high quality, nationally representative household survey data; therefore, the results can be generalized at the national level. Despite this strength, this study has several limitations. Firstly, all information related to ARI symptoms and care seeking was provided by mothers, and was thus subjective in nature. Additionally, the symptoms were not validated by applying any medical examination. As such, there is chance of potential recall bias since retrospective data on their previous events were collected from respondents that may vary in regards to socio-economic status. Furthermore, seasonal variation could not be calculated since data collection was performed at only one point in time. Due to the cross-sectional nature of the present study, it was not possible to identify any causal relationship between ARI and the determining factors.

## Conclusions

ARIs continue to contribute a high disease burden among under-five children in Bangladesh. Factors such as child age, gender, household wealth, and other household lifestyle factors significantly influenced ARI prevalence and care-seeking behaviors. The observed lack of seeking care from medically trained providers is an important concern that suggests policy efforts should take care-seeking patterns into stronger consideration. Higher utilization of formal health care services among educated and wealthier households demonstrated that effective mitigation strategies should employ interventions that particularly target households of low socio-economic status and education level. Based on the results, this study would have an impact on policies for developing effective awareness/health education programs, poverty alleviation program as well as culturally acceptable interventions for improving care-seeking from trained providers, especially for rural residents and should therefore contribute to health system strengthening. It is recommended to improve health care services in terms of their accessibility and affordability through working in partnership with public facilities, private health care activists, and, community-based organizations so that equitable service is ensured.
